# Integrated Action of Rhizobacteria with *Aloe vera* and Moringa Leaf Extracts Improves Defense Mechanisms in *Hibiscus sabdariffa* L. Cultivated in Saline Soil

**DOI:** 10.3390/plants12213684

**Published:** 2023-10-25

**Authors:** Abdel-Raouf Bahgat, Abeer A. Dahab, Abeer Elhakem, Mayank Anand Gururani, Rasha S. El-Serafy

**Affiliations:** 1 Horticulture Department, Faculty of Agriculture, Tanta University, Tanta 31527, Egypt; 2 Medicinal and Aromatic Plants Research Department, Horticulture Research Institute, Agricultural Research Center, Giza 12619, Egypt; dahab.abeer1612@arc.sci.eg; 3 Department of Biology, College of Sciences and Humanities, Prince Sattam Bin Abdulaziz University, Al-Kharj 11942, Saudi Arabia; a.elhakem@psau.edu.sa; 4 Biology Department, College of Science, United Arab Emirates University, Al Ain 15551, United Arab Emirates

**Keywords:** *Hibiscus sabdariffa*, osmotic stress, anthocyanin, rhizobacteria, roselle, secondary metabolites

## Abstract

Osmotic stress is a serious physiological disorder that affects water movement within the cell membranes. Osmotic stress adversely affects agricultural production and sustainability and is largely caused by soil salinity and water stress. An integrated nitrogen-fixing bacteria (NFB) soil amendment and an exogenous foliar application of *Aloe vera* leaf extract (ALE), and moringa leaf extract (MLE) were evaluated on roselle (*Hibiscus sabdariffa* L.) growth, calyx yield, secondary metabolites, and tolerance to osmotic stress in salt-affected soil. The osmotic stress markedly decreased above- and below-ground development of the roselle plant, but integrated NFB soil amendment with ALE or MLE foliar application significantly alleviated its negative impacts. Broadly, an improvement was observed in chlorophyll, carbohydrates, and protein levels following NFB and extracts foliar application, as well as a significant enhancement in antioxidant production (total phenols, ascorbic acid, and FRAP), which decreased peroxide production and increased stress tolerance in plants. Under osmotic stress, the roselle calyx revealed the highest anthocyanin levels, which declined following NFB soil amendment and foliar extract application. Additionally, an enhancement in nitrogen (N), phosphorus (P), and potassium (K) contents and the K/Na ratio, along with a depression in sodium (Na) content, was noticed. The integrated application of *Azospirillum lipoferum* × ALE exhibited the best results in terms of enhancing above- and below-ground growth, calyx yield, secondary metabolites, and tolerance to osmotic stress of the roselle plants cultivated in the salt-affected soil.

## 1. Introduction

*Hibiscus sabdariffa* L. (roselle), an annual plant, is a member of the Malvaceae family and is a commonly used herbaceous plant worldwide. It is cultivated widely throughout Africa, South Asia, and East Asia, as well as in tropical regions all over the world [1]. The calyx, seeds, leaves, and stems of the roselle plant are medicinal, industrial, and food-based, but the calyx’s sepals are the most economically important part [2]. The calyx can contribute to the manufacturing of syrup, tea, wine, jelly, jam, and food coloring [3]. The extract from the calyces shows antioxidant and anticancer properties [4,5] due to its abundant content of anthocyanins, amino and organic acids, vitamin C, β-carotene, flavonoids, steroids, triterpenoids, alkaloids, fats, protein, calcium, and iron [6]. Therefore, roselle plants are exploited as a raw material in the cosmetic and pharmaceutical sectors. The phenolics and anthocyanin in the roselle calyx contain the most active ingredients [7] with medical properties, in abundance. Phenols can scavenge free radicals and act as antioxidants [8]. Anthocyanins have a variety of functions in plants, involving growth, development, and reproduction, in addition to their antioxidant roles in scavenging ROS [9]. Anthocyanin levels usually increase in many plants under saline conditions [10].

Crop production and agriculture are challenged by soil salinity. In the global context, salt-affected soils occupy one billion hectares, and it is predicted that this area will expand due to climate change and poor land and water resource management [11,12]. Soil salinization influences plant growth and development negatively and causes land degradation, leading to a reduction in agricultural output and lowering the economic situation in the region [13]. The harmful influences of soil salinity on crop development are related to the depressed osmotic potential of the soil solution, leading to osmotic, ionic, and oxidative stress, along with nutritional imbalances [14]. If appropriate management strategies or procedures are employed, salt-affected soils can be cultivated [11]. Currently, a wide range of biostimulants are employed to ameliorate the detrimental effects of stress conditions on plants [15,16,17,18] including microorganisms [19].

The nitrogen-fixing bacteria (NFB) as plant growth-promoting rhizobacteria (PGPR) have a direct, positive impact on the physiological and metabolic activities of plant cells, making them environmentally friendly and a sustainable nitrogen source [19]. Moreover, they stimulate plant growth and the synthesis of active substances in medicinal and crop plants under stress conditions [19,20]. NFB provides plants with aerobic nitrogen, released organic growth-stimulant compounds, bioactive compounds, and phytohormones [21,22].

*Aloe vera* is a succulent herb native to Africa and a member of the Liliaceae family. Its leaf produces yellow latex gel [23,24], rich in auxins, gibberellins, salicylic acid, lignin, antioxidants, phenols, flavonoids, amino acids, vitamins, macro- and micronutrients, and polysaccharides [25,26]. The ALE promoted seed germination of *Allium cepa* L. cultivated under saline conditions [27], improved root system development [28], enhanced plant growth, and modified the active ingredients in the geranium plant [29].

Moringa leaf extract (MLE), as a biostimulant with a low cost, environmental friendliness, and as a bioenhancer, improves agricultural sustainability and crop output [18,30]. The MLE is extremely advantageous to plants due to its high content of various phytochemicals, minerals, vitamins, tannins, phenols, and phytohormones [31,32]. The MLE foliar application improved the growth, development, and fruit yield of the pepper plant [33] and enhanced the biomass and yield of the tomato and onion as well as their quantity [34,35]. Similarly, the MLE has been reported to increase salinity stress tolerance in *Phaseolus vulgaris* L. plants [36]. Furthermore, the MLE stimulates the synthesis of active ingredients in medicinal plants under normal and stressful conditions [18].

This study was carried out to evaluate the influence of nitrogen-fixing bacteria strains of *Bacillus polymyxa* (NFB1), *Azotobacter chroococcum* (NFB2), or *Azospirillum lipoferum* (NFB3), as a soil supplementation with foliar spraying with ALE or MLE, on the roots and vegetative growth, fruit attributes, and calyx yield, as well as anthocyanin content, osmoprotectant level, antioxidant activity, and nutrient content of *Hibiscus sabdariffa* L. plants grown in salt-affected soils.

## 2. Results

### 2.1. Root Traits

The length, fresh weight, and dry weight of roselle roots significantly increased following NFB soil application as compared to the un-inoculated controls (Table 1). The least root length (28.2 cm), fresh weight (10.8 g), and dry weight (1.54 g) were exhibited by plants not treated with ALE or MLE foliar spray, whereas ALE treatment significantly induced the greatest root length (33.4 cm), fresh weight (18.1 g), and dry weight (2.35 g), followed by MLE application. The NFB3 × ALE application revealed the highest root fresh weight (25.8 g) and dry weight (2.78 g). On the other hand, control plants grown in salt-affected soil conditions had the lowest root values.

### 2.2. Plant Growth

The height of roselle plants was significantly enhanced following NFB soil application as compared with untreated plants (Table 1). The untreated plants exhibited a negative impact regarding the roselle plant’s height, as the shortest plants (187.9 cm) were observed in untreated plants, whereas the ALE treatment significantly induced the greatest plant height (204.8 cm). In terms of the interaction, the tallest plants (212 cm) were noticed by the NFB3 × ALE application. However, the control plants grown in salt-affected soil conditions had the least value in this respect.

### 2.3. Fruit Attributes and Calyx Yield

**Figure 1:**
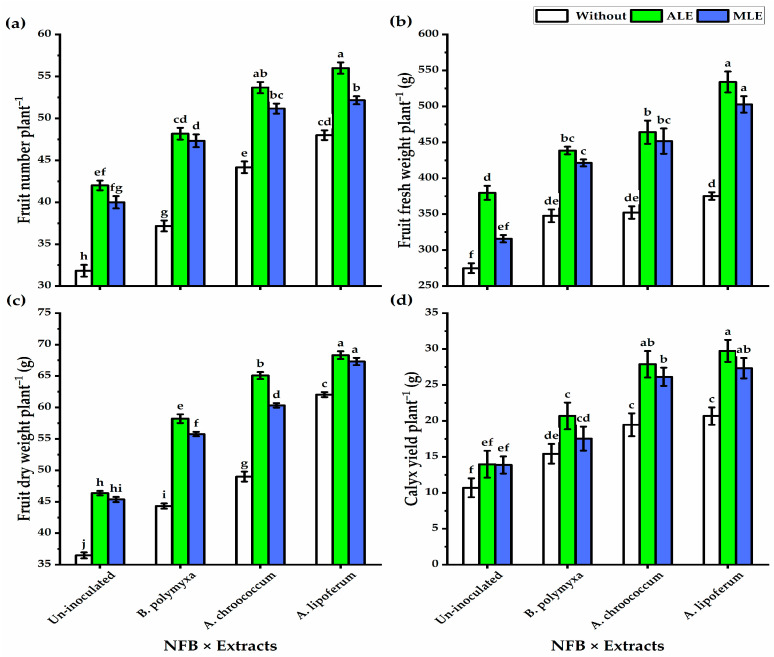
Effect of integrated application of nitrogen-fixing bacteria (NFB) soil supplementation, aloe vera (ALE), and moringa leaf extracts (MLE) foliar application on (**a**) fruit number plant^−1^, (**b**) fruit fresh weight plant^−1^, (**c**) fruit dry weight plant^−1^, and (**d**) calyx yield plant^−1^ of roselle plants cultivated under salt–affected soil. Bars with different letters are significantly different at *p* ≤ 0.05. Values are means ± SE.

Likewise, growth traits, fruit number, fresh and dry weights, as well as calyx yield of the roselle plant showed great enhancement following NFB soil application relative to non-soil applications, and the highest values were revealed by NFB3 soil application, but NFB1 showed the lowest values in this respect (Table 2). Plants not treated with ALE or MLE significantly exhibited the lowest fruit values, but ALE foliar application significantly had the highest fruit number (49.9), fruit fresh (454.1 g), and dry weights (59.07 g), as well as calyx yield per plant^−1^ (23.0 g). The treatment of NFB3 × ALE significantly exhibited the highest fruit traits with the greatest calyx yield plant^−1^ (29.74 g), followed by the treatment of NFB2 × ALE (27.9 g). The lowest calyx yield was recorded by control plants (10.7 g) grown under saline conditions (Figure 1).

### 2.4. Physiological and Biochemical Analysis

#### 2.4.1. Total Chlorophyll, Carbohydrates, and Protein Content

The improvements in plant growth and fruit yield were attributed to an enhancement in chlorophyll content and carbohydrates accumulated in roselle leaves (Table 3). The NFB inoculation and foliar spray with ALE or MLE significantly impacted the leaf chlorophyll content. In this regard, the highest chlorophyll content was related to NFB3 × ALE (0.572 mg g^−1^ FW) and NFB3 × MLE (0.557 mg g^−1^ FW). Additionally, the highest carbohydrate level was given by the NFB3 × ALE treatment, which produced 21% more as compared to untreated plants. Likewise, the maximum protein content obtained by roselle plants subjected to NFB3 × ALE application was 26.3% against 11.3% obtained by untreated plants.

#### 2.4.2. Polyphenol, Ascorbic Acid, and Anthocyanin

The phenolic content in roselle leaves and ascorbic acid in roselle sepals were significantly affected following NFB soil application as compared to the un-inoculated treatment (Table 4), where NFB3 treatment significantly recorded the highest values in this respect. With regard to ALE and MLE foliar applications, the untreated plants exhibited the lowest phenolic content (11.6 mg GAE kg^−1^ DW) and ascorbic acid (56.4 mg 100 g^−1^ DW), whereas ALE treatment significantly recorded the highest phenolics (13.9 mg GAE kg^−1^ DW) and ascorbic acid (61.6 mg 100 g^−1^ DW), levels. The MLE application ranked second in this respect. The highest polyphenol (15.8 mg GAE kg^−1^ DW) and ascorbic acid (65.3 mg 100 g^−1^ DW) contents were given by the NFB3 × ALE application. On the other hand, control plants grown in soil affected by salt had the lowest values. In terms of anthocyanin content, un-inoculated plants recorded the highest anthocyanin (3.68 mg g^−1^ DW), while both NFB treatments and exogenous foliar application reduced its content to reach the lowest following NFB3 × ALE application (2.66 mg g^−1^ DW).

#### 2.4.3. Malondialdehyde and Hydrogen Peroxide Content

With NFB applications, lipid peroxidation (MDA) in the roselle leaves and H_2_O_2_ levels decreased noticeably more than with un-inoculated treatments (Table 5), as NFB3 considerably displayed the lowest values (15.47 mmol g^−1^ FW for MDA and 74.1 Ug g^−1^ FW for H_2_O_2_) in this regard. Moreover, the MDA and H_2_O_2_ readings decreased in both the ALE and MLE applications. NFB3× ALE generated the lowest amounts of MDA and H_2_O_2_ (13.18 mmol g^−1^ FW and 65.2 Ug g^−1^ FW, respectively), whereas the control treatment produced the highest levels in this regard (25.12 mmol g^−1^ FW for MDA and 109.1 Ug g^−1^ FW for H_2_O_2_).

#### 2.4.4. Ferric Ion Reducing Power Assay

The ferric ion-reducing antioxidant power (FRAP) assay in roselle leaves was significantly enhanced following NFB soil application relative to the un-inoculated treatment (Table 5), and the NFB3-treatment significantly produced the highest value in this respect (2.06 mg 100 g^−1^ FW). Concerning ALE and MLE foliar applications, the untreated plants exhibited the lowest values of the FRAP assay (1.87 mg 100 g^−1^ FW), even as the ALE treatment significantly recorded the highest value (2.05 mg 100 g^−1^ FW). The highest FRAP assay (2.19 mg 100 g^−1^ FW) was given by the NFB3 × ALE application. However, control plants grown in soil affected by salt had the lowest values (1.63 mg 100 g^−1^ FW).

#### 2.4.5. Nutrient Content

The results depicted in Table 6 indicate the content of nutrients accumulated in roselle leaves. The content of leaf N, P, and K was significantly enhanced following NFB application as compared with un-inoculated treatments, and NFB3 presented the highest levels in this respect. ALE and MLE foliar applications caused an elevation in nutrient content, reaching its greatest following ALE application.

In terms of the interaction, the treatment of NFB3 × ALE significantly showed the maximum nutrient values in roselle leaves. All NFB applications significantly lowered Na content as compared to the un-inoculated treatments, and the NFB3 treatment significantly gave the lowest Na level (1.55%). Foliar application with ALE and MLE caused a reduction in Na content, reaching its lowest level affected by ALE application. The NFB3 × ALE treatment had the lowest Na content. Meanwhile, the maximum Na level was given by the control treatment. The K/Na ratio was significantly elevated with NFB applications as compared with un-inoculated treatment, which recorded the lowest K and the most elevated Na levels. A significant decrease was noticed with the ALE foliar application. The treatment of NFB3 × ALE significantly presented the highest K:Na ratio (1.85). On the other hand, the control plants significantly exhibited the lowest K:Na ratio (0.09).

## 3. Discussion

Under saline soils, plant roots undergo various morphological changes in their size, diameter, and quantity to enhance nutrition and water absorption as the root density and electrical conductivity increase [37,38]. Plants benefit from the increase in root size through compartmentalization and ion retention [39]. Also, root proliferation contributes to reducing harmful ion accumulation in plants. The osmotic stress negatively impacted roselle growth and development, which consequently had a negative effect on physiological and biochemical traits. The impact of salinity stress is a complex phenomenon that influences several physiological and biochemical processes in plant development, including osmotic stress, ion toxicity, and nutritional imbalance [14]. Excessive salt concentrations prevent plant growth and productivity [40]. This is due to the fact that excessive salt levels contribute to the detrimental cytoplasmic sodium and ion imbalance brought on by drought stress.

The NFB encourages root development and alters the resource-investment strategy of roselle seedlings grown in saline soil. In response to environmental stress, plants may shift more resources to below-ground growth when nitrogen-fixing bacteria are present; this may encourage the development of rhizomes and have an impact on the clonal performance of the plant’s roots [41].

Consequently, interactions with nitrogen-fixing bacteria, especially in saline environments, may contribute to the success of the growth of the roselle plants. Indole-3-acetic acid (IAA)-producing bacteria improve the physicochemical characteristics of soil and enhance the soil quality [42]. All N-fixing bacteria strains used in this study showed significant enhancements in roselle growth and yield under saline conditions. These improvements may be due to the phytohormones produced by N-fixers, including IAA [42], which enhances division, elongation, and differentiation of plant cells [43,44]; tryptophol; and indole butyric acid (IBA), which stimulate plant growth indirectly [45,46]. NFB can increase plant growth by elevating the fixation of atmospheric nitrogen, mineral solubilization, and siderophore production. In this study, fruit attributes appeared to be significantly raised with N-fixers soil amendment, and this may be due to their ability to fix 20–200 kg N ha^−1^, with an increase of about 10–50% of crop yield [47]. Nitrogen (N) is a vital element for plant growth, integrated with chlorophyll, amino acids, proteins, and nucleic acid synthesis, as well as controlling many chemical processes that occur in plant cells and helping electron transportation. It plays an important role in root, stem, and leaf growth and development [48,49,50]. The highest productivity and calyx yield were obtained by *Azospirillum* treatments as compared to *Bacillus* and *Azotobactor* strains, which may be attributed to the detection ability of *Azospirillum* to root exudate components or to the poor adaptation of *Bacillus* and *Azotobactor* strains to root exudates [19]. Increasing antioxidant activity caused a reduction in oxidative stress, which reduced ROS levels under drought and salinity stress [51,52,53]. The N-fixers utilized in this study showed a significant enhancement in the antioxidant content, including FRAP, ascorbic acid, and total phenol, in the roselle plant, along with a reduction in MDA and H_2_O_2_ levels. Similar results were observed by Zhou et al. [16], who stated that NFB soil amendments-maintained membrane integrity and reduced the negative impacts of osmotic stress through increased antioxidant activities and depressed MDA and H_2_O_2_ levels. According to Kumar et al. [54], PGPR stimulated the synthesis of phenolic compounds. Plants are negatively affected by soil salinity and water stress in two ways: first, due to excessive osmotic stress, and second, due to an increase in the accumulation of harmful Na ions [11], as seen in the current study. In this study, saline soil caused an elevation in Na content with a reduction in K content and K/Na ratio in the roselle leaves. Under osmotic stress, the K/Na ratio decreased due to Na toxicity, which inhibits K absorption due to the competition between Na^+^ and K^+^ on the binding sites [55]. Increased K/Na ratios motivate plant tolerance against osmotic stress [56]. NFB showed great potential to reduce Na absorption and elevate P and K uptake. After treatment, an increase in root size and weight was seen, allowing the roselle roots to absorb more K and keep the Na ions away under salt stress to avoid ion toxicity. This reduced the transit of Na into the xylem and isolated Na into the vacuole [57]. Extensive root systems have a characteristic that allows plants to be more effective at absorbing phosphorus [58].

Foliar application with ALE caused an increase in root weight and length of the roselle plants grown in saline soil as compared to the untreated plants. ALE is considered a rich source of auxin, which is a vital component for root growth. At the rooting stage, ALE can be employed as an alternate auxin-enriched in vitro rooting medium for populus plants [28]. The high phosphorus concentration of ALE is essential for the uptake and movement of nutrients, as well as for energy storage and root system development [59]. *Aloe vera* leaf extract is commonly utilized as a natural rooting hormone to help plant cuttings establish new roots [28]. ALE improves root and cell elongation and increases ion transportation [60,61]. Foliar application of ALE significantly boosted plant growth, productivity, and active ingredients, as well as reduced oxidative damage in the roselle leaves cultivated in saline conditions. ALE also significantly increased the calyx yield by 39.3%. *Aloe vera* L. has active compounds of 20 amino acids, 12 vitamins, 20 minerals, and water [62], which contribute to enhancing the plant’s growth. ALE improves oxygen uptake, photosynthesis, respiration, and membrane permeability, leading to an increase in chlorophyll production, the accumulation of more carbohydrates, and greater plant growth [60,61,63]. The theory that ALE contains several physiologically active constituents, such as tryptophan, which is the precursor of auxin, zinc, and endogenous gibberellins, as well as other active compounds to stimulate cell division, may help to explain how ALE has a stimulating effect on the previously vegetative characters [64]. ALE foliar application exhibited an increase in the osmoprotectants (total chlorophyll, phenolics, proteins, and carbohydrates) in the roselle leaves. The increase in growth parameters may be related to ALE’s capacity to stimulate plant development through the assimilation of major and minor nutrients, enzyme activation, modifications of membrane permeability protein synthesis, and stimulation of biomass production [65]. According to Hanafy et al. [66], the direct influence of ALE increases the penetration of molecules into the plant membranes, boosting the accumulation of dry matter and thus increasing the absorption of nutrients. ALE has been employed as a natural plant growth regulator in *Majorana hortensis* and *Salvia officinalis* [59], as well as enhanced the vegetative growth of *Abelmoschuses eculentus*, *Oenothera biennis*, and *Majorana hortensis* [59,67]. A higher anthocyanin content was observed in the calyx of the control, which decreased following ALE foliar application. Anthocyanin accumulation is associated with its reaction to abiotic and biotic stress. The elevation in anthocyanin content in roselle plants can be a strategy for salt tolerance [68]. The oxidative damage that occurred in roselle leaves due to osmotic stress has been alleviated by the ALE application. ALE contains vitamin C and vitamin B complexes, which play an essential role in alleviating stress [62]. ALE foliar supplementation revealed positive effects on boosting antioxidant activity in addition to its beneficial function as a promoter in preserving plant cells from damage caused by oxidative stress through its influence on osmoregulation, protein stability, and antioxidant stability. Thus, the combined application of NFB and ALE caused more improvement in FRAP, ascorbic acid, and total phenols, as well as reduced MDA and H_2_O_2_ content than in solo treatments.

Moringa leaf extract application showed an enhancement in the root traits of the roselle plant. MLE is a potent source of zeatin, which has the function of modifying the expression of specific genes that regulate root growth and root hair elongation [69]. MLE increased the root growth of rape and cabbage plants [70]. Also, MLE enhanced soybean root growth, leading to an increase in the mineralization of nutrients [71]. MLE contributed to improving plant growth, development, and yield attributes, which in turn enhanced crop performance [72,73]. Findings of the current study revealed that under osmotic stress, MLE treatments significantly enhanced plant growth and fruit attributes, as well as reduced the oxidative damage to roselle leaves. Zeatin stimulates food translocation in stem reserves to maintain proper plant physiological and biochemical traits [74], accelerates cytokinin biosynthesis in plants [75], and causes a 30% enhancement in crop productivity and yields [72]. Additionally, it boosts antioxidant content, plant hydration status, and membrane stability [76]. Osmotic stress impedes the plant growth and productivity of roselle plants and markedly destroys the osmolytes, including protein, carbohydrates, ascorbic acid, and chlorophyll. MLE contains several natural stimulants, including proteins, phenolics, amino acids, vitamin E, ascorbates, and other mineral components, which makes it an effective natural growth inducer under stress conditions. Pepper plants showed stimulation in plant growth and yield quality following MLE foliar application [33]. MLE increases the rate of cell division and enlargement, leaf number, and leaf area, causing an increase in photosynthetic traits and yield [77]. Mg, which is an essential element for chlorophyll biosynthesis, is found in MLE in high quantities [78]. The chemical analysis of roselle leaves showed high levels of total phenols and antioxidants. The high level of phenols, vitamins, minerals, and β-carotene in MLE [79] might positively affect the endogenous levels of total phenols and antioxidants in the roselle plant. In the current study, the application of MLE significantly increased the antioxidant levels, leading to higher scavenging activity, which improved the photosynthetic processes and protected the growth of roselle plants from osmotic stress. MLE has a high content of several elements that can enhance the plants’ nutritional deficiencies [80]. According to Abd El-Hamied and El-Amary [81], MLE increased the nutrient content in the leaves of pear trees. MLE has a high content of N, which is essential for maximum crop yields and enhances nutrient absorption by the plant [82]. Foliar application with MLE improved plant vigor, reduced the negative effect of Na, and enhanced plant growth under severe conditions relative to untreated plants [83,84]. The combined application of NFB and MLE may be a beneficial strategy to treat the growing problems of soil salinity and water deficiency.

## 4. Materials and Methods

### 4.1. Study Description and Plant Husbandry

Two field experiments were conducted in the Experimental Farm of Agriculture Faculty, Tanta University, Tanta, Egypt (30°47′18″ N: 31°00′06″ E) at 8 m elevation above sea level during the summer seasons of 2021 and 2022. Before cultivation, samples of the experimental soil were analyzed, and its physical and chemical analyses were analyzed and tabulated in Table 7. Seeds of roselle, *Hibiscus sabdariffa* L. (balady, dark red), were obtained from the Horticulture Research Institute, Medicinal and Aromatic Plants Department, Giza, Egypt. The seeds were planted on the sloping side of rows just above the water line at a 30 cm plant spacing with 60 cm between rows. The seeds were planted on 1 May and 15 April for the first and second seasons, respectively. After 21 days of cultivation, roselle plants were thinned to one plant hill^−1^. All farming practices were performed as recommended.

### 4.2. Nitrogen-Fixing Bacteria

The strains of aerobic nitrogen-fixing bacteria (NFB) of *Bacillus polymyxa* (ATCC 842), *Azotobacter chroococcum* (ATCC 9043), and *Azospirillum lipoferum* (ATCC 29707) were provided from the Soil, Water, and Environment Research Institute, Agricultural Research Center Giza, Egypt, and maintained in the refrigerator at 4 °C until soil supplementation.

### 4.3. Leaf Extract Preparation and Foliar Application

Immediately before application, the aloe leaves were collected from 5-year-old *Aloe vera* plants grown in the Experimental Farm of the Agriculture Faculty, Tanta University, Tanta, Egypt. Aloe gel was extracted from the leaves. After that, ALE was prepared by mixing the gel of the leaves in a house blender. The mixture was watered down to a ratio of 1:30 with distilled water (*v*/*v*). Young leaves of moringa trees were collected from trees grown in the Experimental Farm of Agriculture Faculty, Tanta University, Tanta, Egypt. The leaves were completely mixed with distilled water at a rate of 1:10 *w*/*v* using a house blender [18], then the mixture was filtered using a muslin cloth and watered to a ratio of 1:30 (MLE) with distilled water (*v*/*v*). The surfactant Tween-20 was supplemented at 0.1% (*v*/*v*) to the extracts before application and mixed well. The chemical analysis of ALE and MLE is presented in Table 8.

### 4.4. Treatments and Experimental Design

The current investigation was planned in a split-plot design with twelve treatments in two factors: (1) NFB strains (the NFB0; un-inoculated, NFB1; *Bacillus polymyxa*, NFB2; *Azotobacter chroococcum*, and NFB3; *Azospirillum lipoferum*), and (2) foliar spray extracts (without, ALE, and MLE); each treatment was repeated three times. NFB strains were randomly applied in the main plots, while foliar extracts were supplemented in the subplots. The experimental soil was divided into subplots of 10.5 m^2^ (3.0 × 3.5 m), including five rows, 60 cm between rows, and 35 cm intra-row spacing, with 50 plants per subplot. Bacteria suspension (10 mL) of each strain was individually applied to the experimental main plots as a soil drench 21 days post-germination at a density of 10^9^ cfu ml^−1^. Roselle plants in each subplot were foliar sprayed with ALE or MLE extracts at a volume of 0.2 L three times: 30, 60, and 90 days after cultivation. Untreated control plants received foliar supplementation three times with tap water at the same time of extract application for both seasons.

### 4.5. Growth Attributes

#### 4.5.1. Root Traits

For root determination, roselle seeds were sown on the same day of cultivation in pots (30 cm) filled with the experimental soil. The seedlings were thinned to a seedling/pot. The NFB strains, ALE, and MLE extracts were applied at the same time as the field study application. After 100 days of cultivation, the plants were harvested, and the roots were detached, scrubbed, and washed under running tap water for root length (cm) and fresh and dry weights (g) determination.

#### 4.5.2. Harvesting

Ten plant samples were randomly collected during the harvest stage for the determination of plant height (cm), fruit number plant^−1^, fruit fresh and dry weight plant^−1^ (g), and calyx yield plant^−1^ (g).

### 4.6. Physiological and Biochemical Determinations

For biochemical analysis, at the flowering stage, samples of fresh leaves were picked up and instantly dipped in liquid nitrogen before being squashed to a soft powder and preserved at −80 °C.

#### 4.6.1. Total Chlorophyll

Total chlorophyll (mg g^−1^ FW) of roselle leaves was measured spectrophotometrically at the flowering stage in accordance with Dere’s [85] technique. Fresh leaf samples of 0.2 g were homogenized in 10 mL of 96% methanol for 1 min. The homogenate was filtered before being centrifuged at 2500 rpm for 10 min. The amount of chlorophyll in the supernatant was determined using a UV-VIS spectrophotometer at wavelengths of 666 nm, 653 nm, and 470 nm for chlorophyll a, b, and total carotenoids, respectively.

#### 4.6.2. Total Carbohydrates and Protein Determination

Total carbohydrates (%) in roselle leaves were evaluated using the methods described by Herbert et al. [86]. The phenol solution at 5% was mixed with 1 mL of sugar solution and then 5.0 mL of sulfuric acid. The mixture was thoroughly mixed before being kept in a water bath at 30 °C for 20 min. The UVVIS spectrophotometer was used to measure the produced color at a wavelength of 490 nm. Total protein (%) was determined using the micro-Kjeldahl method, with a nitrogen-to-protein conversion factor of 6.25 [87].

#### 4.6.3. Total Polyphenols, Ascorbic Acid, and Anthocyanin Determination

Polyphenols in the dried leaves were determined using the methods of Dewanto et al. [88] with gallic acid as a standard (mg GAE kg^−1^ DW). Ascorbic acid in sepals (mg 100 g^−1^ DW) was estimated using the method of A.O.A.C. [89]. Anthocyanin content (mg g^−1^ DW) was determined in dried sepals according to the A.O.A.C. [89] method.

#### 4.6.4. Malondialdehyde and Hydrogen Peroxide Estimation

MDA content (mmol g^−1^ FW) was used to determine the amount of lipid peroxidation in roselle leaves, according to Heath and Packer [90]. The H_2_O_2_ content (Ug g^−1^ FW) in roselle leaves was evaluated using the Patterson et al. [91] method.

#### 4.6.5. Ferric Reducing Antioxidant Potential

The FRAP assay in dried leaves was determined calorimetrically according to Benzie and Strain [92]. The FRAP reagent consisted of 20 mM ferric chloride (10:1:1, *v*/*v*/*v*), 10 mM TPTZ (2,4,6-tri-2-pyridyl-1,3,5-triazin), and 300 mM acetate buffer (pH 3.6). A total of 3.0 mL FRAP reagent was mixed with 0.1 mL of methanolic leaf extract and maintained for 8 min at 37 °C. A UV-VIS spectrophotometer was used for FRAP determination at the wavelength of 593 nm. Ascorbic acid was used as a blank sample, and the readings were expressed in mg ascorbic acid equivalent (AAE) per 100 g^−1^ FW.

### 4.7. Nutrient Estimation

For nutrient estimation, a 0.5 g sample of dried leaves was digested using sulfuric and perchloric acids to determine the nutrient content [93,94]. Nitrogen (N) was determined using the micro-Kjeldhl method according to Black et al. [95] and presented as a percentage (%). Phosphorous (P) was assessed colorimetrically as described in the Jackson [94] method using stannous chloride phosphomolibdic-sulforic acid and was calculated in percentages (%). Potassium (K) and sodium (Na) levels were measured by flame photometry and were expressed in percentages (%).

### 4.8. Statistical Analysis

The collected data were statistically analyzed with the MSTAT software, and the combined analysis was performed after conducting Bartlett’s test for the homogeneity of the variances. The Tukey test with a 0.05 probability was used to determine whether there was a significant difference between the mean values [96]. The average means of the two seasons’ results were reported, along with their standard errors (SE) *n* = 10.

## 5. Conclusions

The effects of incorporated NFB soil supplementation and exogenous ALE and MLE foliar application on the growth and productivity of roselle plants cultivated in salt-affected soils were evaluated in this study. NFB with foliar ALE or MLE applications had a marked impact on growth and quality as well as stress tolerance of *Hibiscus sabdariffa* plants, in particular the *Azospirillum lipoferum* × ALE treatment. Dual application of *Azospirillum lipoferum* × ALE significantly increased calyx yield by 155.5% over the control plants, as well as enhanced the active ingredient content, osmotic stress tolerance, and improved nutrient homeostasis in roselle leaves. Our findings indicate that the advantages’ role of *Azospirillum lipoferum* × ALE may be attributed to the activation of the antioxidant defense machinery to alleviate reactive oxygen species (ROS) within stressed plants.

## Figures and Tables

**Table 1:** Root traits and plant height of roselle plants cultivated under salt-affected soil in response to three strains of nitrogen-fixing bacteria (NFB), *Aloe vera* (ALE), and moringa leaf extracts (MLE) and their interaction.

Treatment		Root Length (cm)	Root Fresh Weight (g)	Root Dry Weight (g)	Plant Height (cm)
Nitrogen-fixing bacteria (NFB)					
Un-inoculated (NFB0)		20.7 ± 0.96 c	5.82 ± 0.36 d	1.41 ± 0.08 d	192.0 ± 2.77 b
*Bacillus polymyxa* (NFB1)		29.7 ± 0.57 b	12.08 ± 0.54 c	1.88 ± 0.09 c	191.5 ± 2.04 b
*Azotobacter chroococcum* (NFB2)		37.8 ± 0.62 a	20.30 ± 1.27 b	2.15 ± 0.08 b	195.4 ± 2.03 b
*Azospirillum lipoferum* (NFB3)		36.1 ± 0.62 a	22.39 ± 0.95 a	2.35 ± 0.09 a	207.7 ± 1.88 a
Extracts					
Without		28.2 ± 1.69 c	10.77 ± 1.01 c	1.54 ± 0.079 c	187.96 ± 2.25 c
Aloe leaf extract (ALE)		33.5 ± 1.34 a	18.09 ± 1.59 a	2.35 ± 0.082 a	204.75 ± 1.48 a
Moringa leaf extract (MLE)		31.5 ± 1.30 b	16.58 ± 1.58 b	1.94 ± 0.068 b	197.25 ± 1.74 b
NFB × Extracts					
Un-inoculated (NFB0)	Without	15.6 ± 0.58 g	4.02 ± 0.12 j	0.98 ± 0.02 h	177.5 ± 2.35 f
	ALE	24.5 ± 0.43 ef	7.52 ± 0.16 h	1.74 ± 0.03 f	200.5 ± 2.06 bc
	MLE	22.0 ± 0.68 f	5.93 ± 0.19 i	1.50 ± 0.03 g	198.0 ± 2.16 c
*B. polymyxa* (NFB1)	Without	27.4 ± 0.88 de	9.07 ± 0.09 g	1.46 ± 0.04 g	184.2 ± 1.01 ef
	ALE	31.0 ± 0.87 cd	14.24 ± 0.17 e	2.37 ± 0.04 bc	202.2 ± 2.27 bc
	MLE	30.7 ± 0.49 cd	12.95 ± 0.19 f	1.80 ± 0.04 f	188.2 ± 0.60 de
*A. chroococcum* (NFB2)	Without	35.7 ± 0.88 abc	13.03 ± 0.16 f	1.78 ± 0.06 f	186.7 ± 2.62 def
	ALE	39.8 ± 0.87 a	24.89 ± 0.26 ab	2.51 ± 0.05 b	204.3 ± 1.38 abc
	MLE	37.8 ± 0.83 ab	22.99 ± 0.25 c	2.15 ± 0.05 d	195.2 ± 1.45 cd
*A. lipoferum* (NFB3)	Without	34.1 ± 0.82 bc	16.97 ± 0.33 d	1.95 ± 0.05 e	203.5 ± 2.47 abc
	ALE	38.7 ± 0.70 ab	25.75 ± 0.24 a	2.78 ± 0.05 a	212.0 ± 3.68 a
	MLE	35.5 ± 0.76 abc	24.45 ± 0.31 b	2.32 ± 0.02 c	207.7 ± 3.04 ab
*p*-value					
NFB		<0.001 ***	<0.001 ***	<0.001 ***	<0.001 ***
Extracts		<0.001 ***	<0.001 ***	<0.001 ***	<0.001 ***
NFB × Extracts		<0.05 *	<0.001 ***	0.01 **	<0.01 **

Data (means ± SE, *n* = 10) *, **, and *** indicate, differences at *p* ≤ 0.05, 0.01, and ≤0.01 probability levels, and “ns” indicates a non-significant difference. Mean values sharing the same lower-case letter for NFB, extracts, and their interactions in the same column are not significantly different at *p* ≤ 0.05 from Tukey’s test.

**Table 2:** Fruit traits and calyx yield plant^−1^ of roselle plants cultivated under salt-affected soil in response to three strains of nitrogen-fixing bacteria (NFB), aloe vera (ALE), and moringa leaf extracts (MLE) and their interaction.

Treatment	Fruit Number Plant^−1^	Fruit Fresh Weight Plant^−1^ (g)	Fruit Dry Weight Plant^−1^ (g)	Calyx Yield Plant^−1^ (g)
Nitrogen-fixing bacteria (NFB)				
Un-inoculated	37.94 ± 1.13 d	323.5 ± 11.2 c	42.74 ± 1.09 d	12.85 ± 0.89 d
*Bacillus polymyxa*	44.22 ± 1.27 c	402.6 ± 10.2 b	52.77 ± 1.48 c	17.88 ± 1.03 c
*Azotobacter chroococcum*	49.67 ± 1.04 b	422.7 ± 14.5 b	58.14 ± 1.64 b	24.49 ± 1.23 b
*Azospirillum lipoferum*	52.06 ± 0.85 a	470.6 ± 17.7 a	65.89 ± 0.68 a	25.92 ± 1.19 a
*p*-value	<0.001 ***	<0.001 ***	<0.001 ***	<0.001 ***
Extracts				
Without	40.29 ± 1.34 c	337.6 ± 8.6 c	47.96 ± 1.94 c	16.56 ± 1.04 c
Aloe leaf extract (ALE)	49.96 ± 1.17 a	454.1 ± 12.9 a	59.49 ± 1.76 a	23.07 ± 1.54 a
Moringa leaf extract (MLE)	47.67 ± 1.04 b	422.9 ± 15.1 b	57.20 ± 1.67 b	21.22 ± 1.35 b
*p*-value	<0.001 ***	<0.001 ***	<0.001 ***	<0.001 ***

Data (means ± SE, *n* = 10) *** indicate, differences at *p* ≤ 0.001 probability levels, and “ns” indicates a non-significant difference. Mean values sharing the same lower-case letter for NFB, extracts, and their interactions in the same column are not significantly different at *p* ≤ 0.05 from Tukey’s test.

**Table 3:** Total chlorophyll content (mg g^−1^ FW), total carbohydrate content (%), and total protein content (%) of roselle plants cultivated under salt-affected soil in response to three strains of nitrogen-fixing bacteria (NFB), *Aloe vera* (ALE), and moringa leaf extracts (MLE), and their interaction.

Treatment		Total Chlorophyll (mg g^−1^ FW)	Total Carbohydrates (%)	Total Protein (%)
Nitrogen-fixing bacteria (NFB)				
Un-inoculated		0.353 ± 0.01 d	31.59 ± 0.24 d	15.59 ± 0.78 d
*Bacillus polymyxa*		0.410 ± 0.01 c	32.33 ± 0.27 c	19.73 ± 0.52 c
*Azotobacter chroococcum*		0.467 ± 0.01 b	33.27 ± 0.26 b	22.03 ± 0.67 b
*Azospirillum lipoferum*		0.516 ± 0.02 a	34.78 ± 0.38 a	23.44 ± 0.55 a
Extracts				
Without		0.367 ± 0.01 b	31.68 ± 0.23 c	17.25 ± 0.79 c
Aloe leaf extract (ALE)		0.480 ± 0.02 a	34.39 ± 0.33 a	22.90 ± 0.69 a
Moringa leaf extract (MLE)		0.463 ± 0.02 a	32.91 ± 0.24 b	20.44 ± 0.50 b
NFB × Extracts				
Un-inoculated	Without	0.281 ± 0.01 e	30.41 ± 0.18 g	11.27 ± 0.42 f
	ALE	0.396 ± 0.01 d	32.59 ± 0.18 de	18.28 ± 0.48 de
	MLE	0.383 ± 0.01 d	31.79 ± 0.23 ef	17.22 ± 0.39 e
*B. polymyxa*	Without	0.377 ± 0.01 d	31.15 ± 0.14 fg	17.51 ± 0.56 e
	ALE	0.434 ± 0.01 cd	33.73 ± 0.16 bc	21.70 ± 0.57 bc
	MLE	0.419 ± 0.01 d	32.11 ± 0.18 ef	19.99 ± 0.59 cd
*A. chroococcum*	Without	0.391 ± 0.01 d	32.02 ± 0.14 ef	19.05 ± 0.40 de
	ALE	0.518 ± 0.01 ab	34.48 ± 0.23 b	25.36 ± 0.30 a
	MLE	0.491 ± 0.01 bc	33.32 ± 0.17 cd	21.68 ± 0.60 bc
*A. lipoferum*	Without	0.419 ± 0.01 d	33.15 ± 0.22 cd	21.17 ± 0.24 bc
	ALE	0.572 ± 0.01 a	36.80 ± 0.12 a	26.28 ± 0.47 a
	MLE	0.557 ± 0.01 a	34.40 ± 0.22 b	22.89 ± 0.37 b
*p*-value				
NFB		<0.01 ***	<0.001 ***	<0.001 ***
Extracts		<0.01 ***	<0.001 ***	<0.001 ***
NFB × Extracts		<0.01 **	<0.001 ***	<0.001 ***

Data (means ± SE, *n* = 10) ** and *** indicate, differences at *p* ≤ 0.01 and ≤0.001 probability levels, and “ns” indicates a non-significant difference. Mean values sharing the same lower-case letter for NFB, extracts, and their interactions in the same column are not significantly different at *p* ≤ 0.05 from Tukey’s test. Protein and carbohydrate contents are in the percentage of dry weight.

**Table 4:** Total phenols (mg GAE kg^−1^ DW), ascorbic acid (mg 100 mg^−1^ DW), and anthocyanin (mg g^−1^ DW) of roselle plants cultivated under salt-affected soil in response to three strains of nitrogen-fixing bacteria (NFB), aloe vera (AE), and moringa leaf extracts (MLE) and their interaction.

Treatment		Total Phenols (mg GAE kg^−1^ DW)	Ascorbic Acid (mg 100 mg^−1^ DW)	Anthocyanin (mg g^−1^ DW)
Nitrogen-fixing bacteria (NFB)				
Un-inoculated		11.03 ± 0.17 d	55.50 ± 0.76 d	3.68 ± 0.085 a
*Bacillus polymyxa*		12.44 ± 0.32 c	57.39 ± 0.44 c	3.62 ± 0.088 a
*Azotobacter chroococcum*		13.52 ± 0.29 b	60.18 ± 0.63 b	3.56 ± 0.071 a
*Azospirillum lipoferum*		14.38 ± 0.29 a	63.21 ± 0.58 a	2.81 ± 0.046 b
Extracts				
Without		11.61 ± 0.24 c	56.39 ± 0.69 b	3.73 ± 0.099 a
Aloe leaf extract (ALE)		13.98 ± 0.33 a	61.62 ± 0.60 a	3.11 ± 0.063 c
Moringa leaf extract (MLE)		12.94 ± 0.28 b	59.20 ± 0.71 a	3.41 ± 0.079 b
NFB × Extracts				
Un-inoculated	Without	10.46 ± 0.25 f	51.81 ± 0.21 e	4.10 ± 0.04 a
	ALE	11.71 ± 0.18 ef	59.37 ± 0.26 d	3.32 ± 0.07 de
	MLE	10.92 ± 0.18 f	55.33 ± 0.18 d	3.62 ± 0.06 c
*B. polymyxa*	Without	10.84 ± 0.19 f	55.06 ± 0.13 d	3.97 ± 0.08 a
	ALE	13.74 ± 0.26 bc	59.17 ± 0.22 cd	3.19 ± 0.08 ef
	MLE	12.76 ± 0.20 cde	57.94 ± 0.40 d	3.69 ± 0.06 bc
*A. chroococcum*	Without	12.01 ± 0.23 def	58.46 ± 0.48 d	3.89 ± 0.06 ab
	ALE	14.62 ± 0.25 ab	62.63 ± 0.98 ab	3.29 ± 0.07 de
	MLE	13.92 ± 0.21 bc	59.44 ± 0.94 bc	3.51 ± 0.08 cd
*A. lipoferum*	Without	13.14 ± 0.17 cd	60.23 ± 0.20 d	2.95 ± 0.06 fg
	ALE	15.84 ± 0.20 a	65.32 ± 0.65 a	2.66 ± 0.04 g
	MLE	14.16 ± 0.21 bc	64.09 ± 0.38 a	2.82 ± 0.08 g
*p*-value				
NFB		<0.001 ***	<0.001 ***	<0.001 ***
Extracts		<0.001 ***	<0.001 ***	<0.001 ***
NFB × Extracts		<0.01 **	<0.01 **	<0.01 **

Data (means ± SE, *n* = 10) ** and *** indicate, differences at *p* ≤ 0.01 and ≤0.001 probability levels, and “ns” indicates a non-significant difference. Mean values sharing the same lower-case letter for NFB, extracts, and their interactions in the same column are not significantly different at *p* ≤ 0.05 from Tukey’s test.

**Table 5:** MDA (mmol g^−1^ FW), H_2_O_2_ (Ug g^−1^ FW), and FRAP (mg 100 g^−1^ DW) of roselle plants cultivated under salt-affected soil in response to three strains of nitrogen-fixing bacteria (NFB), *Aloe vera* (AE), and moringa leaf extracts (MLE) and their interaction.

Treatment		MDA (mmol g^−1^ FW)	H_2_O_2_ (Ug g^−1^ FW)	FRAP (mg 100 g^−1^ FW)
Nitrogen-fixing bacteria (NFB)				
Un-inoculated		23.17 ± 0.39 a	103.0 ± 1.2 a	1.81 ± 0.04 d
*Bacillus polymyxa*		21.66 ± 0.26 b	86.9 ± 1.9 b	1.94 ± 0.01 c
*Azotobacter chroococcum*		17.46 ± 0.55 c	79.8 ± 1.1 c	2.01 ± 0.03 b
*Azospirillum lipoferum*		15.47 ± 0.62 d	74.1 ± 1.7 d	2.06 ± 0.03 a
Extracts				
Without		21.69 ± 0.53 a	92.7 ± 2.4 a	1.87 ± 0.04 c
Aloe leaf extract (ALE)		17.74 ± 0.78 c	79.3 ± 2.6 c	2.05 ± 0.03 a
Moringa leaf extract (MLE)		18.89 ± 0.71 b	85.9 ± 2.0 b	1.94 ± 0.01 b
NFB × Extracts				
Un-inoculated	Without	25.12 ± 0.41 a	109.1 ± 1.07 a	1.63 ± 0.07 d
	ALE	21.52 ± 0.25 c	98.4 ± 0.52 bc	1.93 ± 0.03 c
	MLE	22.86 ± 0.25 b	101.6 ± 1.12 b	1.86 ± 0.02 c
*B. polymyxa*	Without	22.70 ± 0.41 b	97.0 ± 0.87 c	1.90 ± 0.02 c
	ALE	20.99 ± 0.31 cd	78.9 ± 1.10 fg	1.97 ± 0.02 bc
	MLE	21.31 ± 0.28 c	84.6 ± 1.17 d	1.93 ± 0.02 c
*A. chroococcum*	Without	20.15 ± 0.30 d	83.9 ± 0.84 de	1.96 ± 0.04 bc
	ALE	15.26 ± 0.63 g	74.4 ± 0.90 h	2.10 ± 0.06 ab
	MLE	16.96 ± 0.28 f	80.9 ± 0.97 ef	1.98 ± 0.03 bc
*A. lipoferum*	Without	18.81 ± 0.31 e	80.5 ± 1.87 ef	1.99 ± 0.03 bc
	ALE	13.18 ± 0.48 h	65.2 ± 1.05 i	2.19 ± 0.06 a
	MLE	14.43 ± 0.26 g	76.7 ± 1.02 gh	2.00 ± 0.03 bc
*p*-value				
NFB		<0.001 ***	<0.001 ***	<0.001 ***
Extracts		<0.001 ***	<0.001 ***	<0.001 ***
NFB × Extracts		<0.001 ***	<0.001 ***	<0.01 **

Data (means ± SE, *n* = 10) ** and *** indicate, differences at *p* ≤ 0.01 and ≤0.001 probability levels, and “ns” indicates a non-significant difference. Mean values sharing the same lower-case letter for NFB, extracts, and their interactions in the same column are not significantly different at *p* ≤ 0.05 from Tukey’s test.

**Table 6:** Mineral content N, P, K, Na, and K: Na ratio of roselle plants cultivated under salt-affected soil in response to three strains of nitrogen-fixing bacteria (NFB), *Aloe vera* (AE), and moringa leaf extracts (MLE) and their interaction.

Treatment		N (%)	P (%)	K (%)	Na (%)	K:Na Ratio
Nitrogen-fixing bacteria (NFB)						
Un-inoculated		2.49 ± 0.13 d	0.853 ± 0.01 d	1.97 ± 0.03 c	1.81 ± 0.04 a	1.10 ± 0.03 d
*Bacillus polymyxa*		3.16 ± 0.08 c	0.94 ± 0.02 c	2.11 ± 0.02 b	1.68 ± 0.04 b	1.27 ± 0.04 c
*Azotobacter chroococcum*		3.53 ± 0.11 b	1.03 ± 0.02 b	2.17 ± 0.04 b	1.58 ± 0.03 c	1.39 ± 0.05 b
*Azospirillum lipoferum*		3.75 ± 0.09 a	1.12 ± 0.03 a	2.32 ± 0.05 a	1.55 ± 0.04 c	1.52 ± 0.07 a
Extracts						
Without		2.76 ± 0.13 c	0.89 ± 0.02 b	2.00 ± 0.02 c	1.81 ± 0.03 a	1.12 ± 0.03 c
Aloe leaf extract (ALE)		3.66 ± 0.11 a	1.05 ± 0.03 a	2.28 ± 0.04 a	1.50 ± 0.02 c	1.54 ± 0.05 a
Moringa leaf extract (MLE)		3.27 ± 0.08 b	1.01 ± 0.03 a	2.14 ± 0.03 b	1.65 ± 0.02 b	1.31 ± 0.03 b
NFB × Extracts						
Un-inoculated	Without	1.80 ± 0.07 f	0.81 ± 0.01 h	1.84 ± 0.02 d	2.01 ± 0.04 a	0.92 ± 0.02 h
	ALE	2.92 ± 0.08 de	0.89 ± 0.01 efgh	2.06 ± 0.02 bc	1.70 ± 0.01 c	1.21 ± 0.01 efg
	MLE	2.76 ± 0.06 e	0.87 ± 0.01 fgh	1.99 ± 0.02 cd	1.72 ± 0.02 c	1.16 ± 0.02 fg
*B. polymyxa*	Without	2.80 ± 0.09 e	0.84 ± 0.01 gh	2.01 ± 0.02 cd	1.87 ± 0.0 b	1.08 ± 0.01 gh
	ALE	3.47 ± 0.09 bc	1.01 ± 0.02 bcde	2.20 ± 0.04 b	1.52 ± 0.02 ef	1.45 ± 0.04 c
	MLE	3.20 ± 0.09 cd	0.97 ± 0.01 cdefg	2.12 ± 0.02 bc	1.64 ± 0.03 cd	1.29 ± 0.03 cdef
*A. chroococcum*	Without	3.05 ± 0.06 de	0.94 ± 0.01 defgh	2.05 ± 0.02 bc	1.73 ± 0.02 c	1.19 ± 0.02 efg
	ALE	4.06 ± 0.05 a	1.10 ± 0.04 abc	2.35 ± 0.02 a	1.45 ± 0.04 fg	1.63 ± 0.04 b
	MLE	3.47 ± 0.10 bc	1.05 ± 0.03 bcd	2.09 ± 0.02 bc	1.56 ± 0.03 de	1.34 ± 0.03 cde
*A. lipoferum*	Without	3.39 ± 0.04 bc	1.00 ± 0.04 cdef	2.09 ± 0.03 bc	1.64 ± 0.04 cd	1.28 ± 0.04 def
	ALE	4.20 ± 0.08 a	1.21 ± 0.02 a	2.49 ± 0.06 a	1.35 ± 0.02 g	1.85 ± 0.06 a
	MLE	3.66 ± 0.06 b	1.14 ± 0.04 ab	2.36 ± 0.04 a	1.67 ± 0.04 cd	1.42 ± 0.05 cd
*p*-value						
NFB		<0.001 ***	<0.001 ***	<0.001 ***	<0.001 ***	<0.001 ***
Extracts		<0.001 ***	<0.001 ***	<0.001 ***	<0.001 ***	<0.001 ***
NFB × Extracts		<0.001 ***	ns 0.576	<0.01 **	<0.001 ***	<0.001 ***

Data (means ± SE, *n* = 10) ** and *** indicate, differences at *p* ≤ 0.01 and ≤0.001 probability levels, and “ns” indicates a non-significant difference. Mean values sharing the same lower-case letter for NFB, extracts, and their interactions in the same column are not significantly different at *p* ≤ 0.05 from Tukey’s test.

**Table 7:** The physio-chemical properties of the experimental soil.

Soil Characteristics								Cations				Anions		
	Sand	Silt	Clay	pH	EC	ESP	OM	Na^+^	Ca^2+^	K	Mg^2+^	HCO^3−^	SO_4_^2−^	Cl^2−^
	%	%	%		dSm^−1^	%	%	meq L^−1^				meq L^−1^		
Season 1	65.24	13.14	21.62	7.9	4.9	15.9	1.15	30.93	10.67	0.456	7.01	5.52	18.94	24.61
Season 2	64.87	13.66	21.47	8.1	4.4	15.5	1.28	26.86	9.20	0.46	7.49	5.73	18.82	22.09

ESP: exchangeable sodium percentage, OM: organic matter.

**Table 8:** Chemical analysis of *Aloe vera* and moringa leaf extracts.

*Aloe vera*		Moringa	
Component	Value	Component	Value (mg g^−1^ DW)
Total phenols	30.82 µg g^−1^	Total chlorophyll	3.86 mg g^−1^ DW
Total sugars	12%	Total carotenoids	1.65 mg g^−1^ DW
Total protein	2.82 mg g^−1^	Total phenols	1.704 mg g^−1^ DW
Antioxidant activity	41.8%	Total sugars	346.16 mg g^−1^ DW
Ascorbic acid	154.64 mg g^−1^ FW	Ascorbic acid	9.28 mg g^−1^ FW
Nutrients value (mg 100 mL^−1^ FW)		Nutrients value (mg g^−1^ DW)	
Nitrogen	89	Nitrogen	13.23
Phosphorus	8	Phosphorus	3.18
Potassium	67	Potassium	12.45
Magnesium	16	Magnesium	2.98
Calcium	33	Calcium	17.08
Iron	0.5	Iron	0.412

## Data Availability

Data are contained within the article.
